# The Efficacy and Complications of Deep Sedation in Pediatric Dental Patients: A Retrospective Cohort Study

**DOI:** 10.1155/2022/5259283

**Published:** 2022-06-22

**Authors:** Seyed Sajad Razavi, Bita Malekianzadeh

**Affiliations:** ^1^School of Medicine, Shahid Beheshti University of Medical Sciences, Tehran, Iran; ^2^School of Medicine, Tehran University of Medical Sciences, Tehran, Iran

## Abstract

**Background:**

Dental anxiety in children is a common problem. Currently, many of dental procedures are performed under sedation. Different methods of sedation have been employed for this purpose. Compared to adults, children usually need a deeper sedation level. The aim of this retrospective study is to assess the efficacy and complication of deep sedation in pediatric dental patients.

**Method:**

This study was performed on 250 ASA (American Society of Anesthesiologists) I, II children undergoing deep sedation during the dental procedures. After the administration of oral midazolam as premedication, the monitoring process started. The patients that received the sedation dose of propofol and oxygen through nasal cannula during the procedure were carefully monitored for the purpose of evaluating hemodynamic and respiratory complications. The mean procedure and recovery time, postoperative nausea and vomiting (PONV), and success rate were further studied.

**Result:**

The average age of the patients was 3.7. 32% of the patients were females, and 68% of them were males. Laryngospasm that occurred in 5 cases was resolved immediately by using positive pressure ventilation. Mild hypoxia was observed in 17 cases which were immediately managed by a bag-valve-mask ventilation. No cases of hemodynamic complications and PONV were reported. The mean length of the procedure was 57 minutes, and the mean length of recovery was 16 minutes. The success rate of this method was estimated to be 99.6%.

**Conclusion:**

Deep sedation with propofol is a suitable technique with a high success rate for dental procedures in children. It was also concluded that in pediatric dental procedures, the presence of a skilled anesthetist and the implementation of a close monitoring process are required.

## 1. Introduction

In recent years, outpatient procedures such as dental treatments in children have tremendously increased. Childhood caries is the most common chronic disease in children. Dental fear and anxiety are common in children, especially in children with behavioral problems. Intense anxiety not only causes complications for the patient but also reduces the quality of the dentist's performance. Controlling the child's fear and pain is an important factor that plays a pivotal role in ensuring the pediatric patient's immobility and increasing the quality of the dentist's work. To these ends, various sedation techniques have been used for the purpose of dental treatments in children. [1–3].

Conscious sedation or moderate sedation is a level of sedation at which the airway protective reflexes are intact and the patient's consciousness is maintained. This technique is used in adults and older children who are cooperative for the purpose of relieving their tension. In moderate sedation, the patients are sleepy though able to follow the dentist's instructions. Patients breathe well, and they wake up easily. The cardiovascular function is also preserved. Although moderate sedation is unpredictable in young children, it is often used successfully in older children. It is not uncommon that deep sedation state changes to general anesthesia as a complication. The patient may be unable to maintain airway patency, and breathing may be compromised. The patient is fully asleep and could not wake up easily. According to the AAPD (American Association of Pediatric Dentistry) guidelines, deep sedation should be only performed by a qualified health professional accredited for this purpose [4, 5].

Presedation evaluation is required for all types of sedation including the diagnosis of airway problems such as tonsillar hypertrophy, fasting guidelines, and available pediatric anesthesia equipment. Sedation for dentistry operation purposes is not without risk, which can be reduced by improving preoperative conditions. General anesthesia is recommended for children under 4, ASA III and higher patients, mouth breathers as well as patients suffering from physiologic and mental problems. Airway complications such as apnea, hypoxia, laryngospasm, and bronchospasm are more likely to occur in young children during the sedation process [6, 7]. However, due to the lack of cooperation and the patients' high anxiety level, light and moderate sedation could not be practical in young children.

There are oral, nasal, intramuscular, intravenous, subcutaneous, and inhalation routes for administering sedation to children. Propofol is an intravenous anesthetic agent with rapid onset and recovery time. The propofol subanesthetic dose is used for conscious sedation in older children. It fails to have an analgesic effect. Therefore, in painful procedures, ketofol (ketamine + propofol) is recommended. The practitioner who uses propofol must be trained in airway management as well as basic and advanced life support. The required proper monitoring and emergency airway equipment should be available. The advantages of propofol for outpatient procedures include less postoperative nausea and vomiting, and rapid recovery [8, 9]. Studies on the efficacy and complications of various sedation techniques showed their inadequacy in pediatric dental patients. In addition, most of the studies in the literature focused on moderate sedation [10]. A number of neurologic damages and death cases have been reported in children who were sedated for dental procedures. In pediatric dentistry, patient sedation must be safely performed [4, 11]. Accordingly, the present study evaluates the efficacy and safety of deep sedation with propofol infusion in 250 pediatric patients.

## 2. Method

All of the patients were ASA (American Society of Anesthesia) I, II. The patients that underwent deep sedation in the dental office of a pediatric hospital were included in this study. The data from the patients' records were extracted. Parental consent was obtained to review the information. All of the patients were eligible for sedation, and presedation evaluation was done. Oral midazolam (0.5 mg/kg) was prescribed for all of the patients. Then, the intravenous catheter was inserted in 15 minutes. Subsequently, after establishing the monitoring process, electrocardiogram (ECG), noninvasive blood pressure monitoring (NIBP), and pulse oximetry, we injected propofol 2 mg/kg due to the need for unresponsiveness to environmental stimuli. In adults, the recommended sedation dose of propofol as a loading dose is 0.5–1 mg/kg. However, in pediatric patients, higher doses will be implemented. All of the patients received oxygen with nasal cannula and intravenous propofol infusion 75–100 *µ*g/kg/min during the procedure. The sedation was performed by the pediatric anesthesiologist, and the pediatric dentist blocked nerve conduction by the local anesthetic. The patients were sedated deeply and had spontaneous ventilation. Propofol infusion dose was adjusted according to the undersedation or oversedation condition. The procedure duration, and airway and breathing complications including hypoxia (SpO2 < 94%), laryngospasm: complete or partial, bronchospasm, and apnea were recorded. Furthermore, the procedure success, namely, the number of cases in which the dental procedure was performed completely under sedation relative to the total number of patients, postoperative nausea and vomiting, and recovery were considered. The anesthesiologist closely monitored the patients. The diagnosis of laryngospasm and apnea was made clinically by the anesthesiologist. The same pediatric anesthesiologist was delivering care in all cases. The data were collected from the patients' records. The patients' information was exactly recorded for possible investigation. The data were processed and calculated using Excel software.

## 3. Results

The patients' age was ranged between 2.5 and 5. The average age of the patients was 3.7 years. Laryngospasm, partial with stridor, occurred in 5 cases (2%) that was resolved by using positive pressure ventilation with fiO_2_ 100%. None of the patients had complete laryngospasm. In 17 cases (6.8%), mild hypoxia (SpO2:90–94%) happened which was immediately managed with bag mask ventilation. In all cases of laryngospasm and hypoxia, oropharyngeal secretion suctioning and airway opening maneuvers including jaw thrust were performed before using bag mask or positive pressure ventilation. The procedure was interrupted and canceled in one case due to repeated oxygen desaturation. By further investigation, we diagnosed adenoid hypertrophy. None of the patients required additional interventions such as nasopharyngeal airway or intubation. Hemodynamics of the patients was stable during the sedation, and bradycardia (heart rate less than 60/min) or hypotension (SBP< 90 mmHg) did not occur. No case of PONV was reported. The mean procedure time was 57 minutes, and the mean recovery time was 16 minutes. The success rate of this method was estimated to be 99.6%.

## 4. Discussion

The number of operating rooms for pediatric dentistry purposes has increased over the last decades. Lack of cooperation or long-term procedure often leads to sedation or general anesthesia. Dental procedures are common in pediatric dentistry, and the patient's fear must be controlled to ensure both safety and quality [12, 13]. Sedation in children is different from adults. In addition to ensuring anxiolysis and analgesia, sedation should lead to immobility in this group. Studies have shown that compared to adults, the possibility of changing the intended sedation level to a deeper level in children is higher. Practitioners should recognize this problem and manage it. Currently, there are scant data on the mortality and morbidity induced by anesthesia and sedation, especially deep sedation, in pediatric dental procedures. Previous studies demonstrated that the highest mortality rate is observed in 2- to 5-year-old children, in office-based dentistry operations, and in pediatric procedures operated by the untrained personnel. Overall, children younger than 6 are more susceptible to life-threatening complications including apnea, airway obstruction, bronchospasm, and laryngospasm. The present study evaluated the efficacy and safety of deep sedation with propofol in pediatric dentistry. In this study, it was further shown that deep sedation with propofol infusion in the presence of an anesthesiologist and close monitoring are both effective and safe [14–16]. According to the AAPD (American Association of Pediatric Dentistry) guidelines, during deep sedation, the presence of two trained practitioners is necessary. These practitioners must be experts in patient monitoring, drug administration, airway management, and basic and advanced life support. They must be ready for resolving issues during the emergency events [7, 17]. In the present study, pediatric anesthesiologist carried out sedation, and an anesthesia nurse monitored all the patients continuously. The presence of a dentist anesthesiologist for providing office-based sedation is an emerging trend in the United States of America [18, 19].

Most of the previous studies concentrate on conscious sedation in dentistry. There is inadequate evidence regarding the efficacy of oral midazolam or nitrous oxide [20]. Postdischarge complications after deep sedation with propofol were low [21]. The risk of long sleep, irritability, and nausea was higher due to multidrug sedation with hydroxyzine, meperidine, and chloral hydrate [22–25].

In one study, deep sedation with propofol + ketamine was compared to propofol + fentanyl in a hospital-based pediatric dental office. All of the procedures were successfully performed. Mild hypoxia occurred in 24% of the patients which was resolved by using nasal cannula oxygenation [12].

In the present study, airway complications were not high which could be due to the presence of a pediatric anesthesiologist as well as close observation and monitoring. Although the prevalence of laryngospasm with propofol is generally lower than other anesthetic drugs, dental procedures due to proximity to the airway can affect the process. The limitations of this study include the absence of a control group for between-group comparisons, and end tidal CO_2_ and bispectral index (BIS) monitoring. The total dose of consumed propofol was not calculated as well. Future studies could address the efficacy and safety of deep sedation with different drugs in young children. The presence of expert practitioners and careful monitoring for deep sedation is necessary.

## 5. Conclusion

Deep sedation with propofol is a suitable technique with a high success rate for dental procedures in children. The presence of a skilled anesthetist and close monitoring are essential. Without careful observation and monitoring, this procedure could be risky.

## Data Availability

The data of this study are available in medical records of the Department of Mofid Children Hospital.

## References

[1] Attri J. P., Sharan R., Makkar V., Gupta K. K., Khetarpal R., Kataria A. P. (2017). Conscious sedation: emerging trends in pediatric dentistry. *Anesthesia, Essays and Researches*.

[2] Rosenberg M. (2021). *Pediatric Sedation Outside of the Operating Room*.

[3] Lee H., Milgrom P., Huebner C. E. (2017). Ethics rounds: death after pediatric dental anesthesia: an avoidable tragedy?.

[4] Nelson T. M., Xu Z. (2015). Pediatric dental sedation: challenges and opportunities.Clinical. *Cosmetic and Investigational Dentistry*.

[5] (2018). Practice Guidelines for Moderate Procedural Sedation and Analgesia 2018:report by the american society of anesthesiologists task force on moderate procedural sedation and analgesia, the american association of oral and maxillofacial surgeons, american college of radiology, american dental association, american society of dentist anesthesiologists, and society of interventional radiology. https://pubmed.ncbi.nlm.nih.gov/29334501/.

[6] Ba’akdah R., Farsi N., Boker A., Al Mushayt A. (2008). The use of general anesthesia in pediatric dental care of children at multi-dental centers in Saudi Arabia.

[7] Coté Cj Fau - Wilson S., Wilson S. (2019). Guidelines for monitoring and management of pediatric patients before, during, and after sedation for diagnostic and therapeutic procedures. *Hong Kong Medical Journal*.

[8] Chiaretti A., Benini F., Pierri F. (2014). Safety and efficacy of propofol administered by paediatricians during procedural sedation in children. *Acta Paediatrica*.

[9] Jain R. (2013). Efficacy and safety of deep sedation by non-anesthesiologists for cardiac mri in children. *Pediatric Radiology*.

[10] Tobias J. D., Leder M. (2011). Procedural sedation: a review of sedative agents, monitoring, and management of complications. *Saudi Journal of Anaesthesia*.

[11] Lee H. H. (2013). Trends in death associated with pediatric dental sedation and general anesthesia. *Paediatric anaesthesia*.

[12] Ahmed S. S., Hicks S. R., Slaven J. E., Nitu M. E. (2016). Deep sedation for pediatric dental procedures: is this a safe and effective option?. *The Journal of Clinical Pediatric Dentistry*.

[13] Lera dos Santos M. E., Maluf-Filho F., Chaves D. M. (2013). Deep sedation during gastrointestinal endoscopy: propofol-fentanyl and midazolam-fentanyl regimens. *World journal of gastroenterology*.

[14] Motas D., McDermott N. B., VanSickle T., Friesen R. H. (2014). Depth of consciousness and deep sedation attained in children as administered by nonanaesthesiologists in a children’s hospital. *Paediatr Anaesth*.

[15] Cravero J. P., Beach M. L., Blike G. T., Gallagher S. M., Hertzog J. H. (2004). Incidence and nature of adverse events during pediatric sedation/anesthesia for procedures outside the operating room: report from the pediatric sedation research consortium. *International Anesthesia Research Society*.

[16] Vetri Buratti C., Angelino F., Sansoni J., Fabriani L., Mauro L., Latina R. (2015). Distraction as a technique to control pain in pediatric patients during venipuncture a narrative review of literature. *Professioni Infermieristiche*.

[17] (2012). Guideline on use of anesthesia personnel in the administration of office-based deep sedation/general anesthesia to the pediatric dental patient. *Pediatric dentistry*.

[18] Olabi N. F., Jones J. E., Saxen M. A., Sanders B. J., Walker L. A. (2013). The use of office-based sedation and general anesthesia by board certified pediatric dentists practicing in the united states. *Anesthesia Progress*.

[19] Tarver M., Fau - Primosch R. G. M., Primosch R. (2012). impact of office-based intravenous deep sedation providers upon traditional sedation practices employed in pediatric dentistry. *American Academy of Pedodontics*.

[20] Jones R. (2012). Weak evidence that oral midazolam is an effective sedative agent for children undergoing dental treatment. *Evidence-based dentistry*.

[21] Davidovich E., Meltzer L., Efrat J., Gozal D., Ram D. (2017). Post-discharge events occurring after dental treatment under deep sedation in pediatric patients. *The Journal of clinical pediatric dentistry*.

[22] Martinez D., Wilson S. (2006). Children sedated for dental care: a pilot study of the 24-hour postsedation period. *Pediatric Dentistry*.

[23] McCormack L., Chen J. W., Trapp L., Job A. (2014). A comparison of sedation-related events for two multiagent oral sedation regimens in pediatric dental patients. *American Academy of Pedodontics*.

[24] Ritwik P., Cao L. T., Curran R., Musselman R. J. (2013). Post-sedation events in children sedated for dental care. *Nesthesia Progress*.

[25] Stamp A. J., Rolland S. L., Wilson K. E., Vernazza C. R. (2019). Conscious sedation in children: the need to strengthen the evidence base remains. *Evidence-Based Dentistry*.

